# Phenotypic and genomic survey on organic acid utilization profile of *Pseudomonas mendocina* strain S5.2, a vineyard soil isolate

**DOI:** 10.1186/s13568-017-0437-7

**Published:** 2017-06-26

**Authors:** Teik Min Chong, Jian-Woon Chen, Wah-Seng See-Too, Choo-Yee Yu, Geik-Yong Ang, Yan Lue Lim, Wai-Fong Yin, Catherine Grandclément, Denis Faure, Yves Dessaux, Kok-Gan Chan

**Affiliations:** 1 Division of Genetics and Molecular Biology, Institute of Biological Sciences, Faculty of Science, University of Malaya, 50603 Kuala Lumpur, Malaysia; 2 UM Omics Centre, University of Malaya, 50603 Kuala Lumpur, Malaysia; 3 Integrative Pharmacogenomics Institute (iPROMISE), Universiti Teknologi MARA, 40450 Shah Alam, Selangor Malaysia; 4 Institute for Integrative Biology of the Cell (I2BC), CEA, CNRS, Université Paris-Sud, Université Paris-Saclay, 91198 Gif-Sur-Yvette, France

**Keywords:** *Pseudomonas mendocina*, Single molecule real-time (SMRT) sequencing, Vineyard soil, Grapevine exudates, Organic acids, Carbon utilization enzymes

## Abstract

Root exudates are chemical compounds that are released from living plant roots and provide significant energy, carbon, nitrogen and phosphorus sources for microbes inhabiting the rhizosphere. The exudates shape the microflora associated with the plant, as well as influences the plant health and productivity. Therefore, a better understanding of the trophic link that is established between the plant and the associated bacteria is necessary. In this study, a comprehensive survey on the utilization of grapevine and rootstock related organic acids were conducted on a vineyard soil isolate which is *Pseudomonas mendocina* strain S5.2. Phenotype microarray analysis has demonstrated that this strain can utilize several organic acids including lactic acid, succinic acid, malic acid, citric acid and fumaric acid as sole growth substrates. Complete genome analysis using single molecule real-time technology revealed that the genome consists of a 5,120,146 bp circular chromosome and a 252,328 bp megaplasmid. A series of genetic determinants associated with the carbon utilization signature of the strain were subsequently identified in the chromosome. Of note, the coexistence of genes encoding several iron–sulfur cluster independent isoenzymes in the genome indicated the importance of these enzymes in the events of iron deficiency. Synteny and comparative analysis have also unraveled the unique features of d-lactate dehydrogenase of strain S5.2 in the study. Collective information of this work has provided insights on the metabolic role of this strain in vineyard soil rhizosphere.

**Electronic supplementary material:**

The online version of this article (doi:10.1186/s13568-017-0437-7) contains supplementary material, which is available to authorized users.

## Introduction

Root exudates are rhizodeposits that are released from living plant roots into the surrounding rhizosphere (Uren 2007). These compounds mainly consisted of water-soluble sugars, organic acids, and amino acids, providing significant energy sources for microbes inhabiting the rhizosphere and its vicinity (Brimecombe et al. 2001). This represents a form of plant–microbe interaction that enables colonization and development of active microbial populations in plant roots and the surrounding soil (Bais et al. 2006; Haichar et al. 2008; Nihorimbere et al. 2011). Although the nature of exudates varies according to growth stages of a given plant, the composition of root exudates is also influenced by environmental factors such as pH, temperature, availability of nutrients, and presence of microorganisms (Nihorimbere et al. 2011; Singh and Mukerji 2006). Furthermore, the differences in exudation profiles directly impact the composition of the microflora inhabiting the specific niche that the rhizosphere is (Singh and Mukerji 2006; Mondy et al. 2014). The exudates shape the microflora associated with the plant, and further influences plant health and productivity, hence a better understanding of the trophic link that is established between the plant and the associated bacteria is necessary.

Grapevine (*Vitis vinifera* L.) is a non-climacteric fruit crop that grows as deep-rooted perennial plant (Archana et al. 2011). *Pseudomonas* spp. namely *Pseudomonas fluorescens*, *P*. *lini*, *P*. *mendocina*, *P*. *putida*, and *P*. *syringae* were among the soil inhabitants commonly found at both the acidic and alkaline soils of these native grapevines (Chan et al. 2016; Chenier et al. 2008; Chong et al. 2012, 2016; Karagöz et al. 2012). Our previous investigation on microbiota inhabiting the vineyard soil in Riquewihr, France has led to the isolation of *P. mendocina* strain S5.2 that harbor resistance traits towards various heavy metals (Chong et al. 2012).

In this study, a further elucidation of utilization of grapevine related compounds was conducted to gain insight on the intricate interaction occurring between the strain and the grapevine. Our work aimed at inclusively determining the phenotypic and genomic profiles associated with grapevine exudate utilization. With reference to the collective reports of various organic acids detected from grapevine and rootstock related exudates, a gene-trait matching approach followed by a comparative analysis was employed to unravel the complete profile of genetic determinants associated with the displayed utilization of the carbon compounds by this strain.

## Materials and methods

### Isolation and identification of bacteria

*Pseudomonas mendocina* strain S5.2 was isolated from a vineyard soil in Riquewihr, in the Alsace region of France. Isolation of this strain was performed using KG minimal medium as previously described (Chong et al. 2016). Routine maintenance of the culture was performed on Luria–Bertani (LB) (Merck, NJ, USA) medium at 28 °C.

Identification of the strain was conducted via 16S rRNA gene sequencing followed by phylogenetic analysis and pairwise similarity analysis using EzBioCloud database (http://www.ezbiocloud.net/identify) (Kim et al. 2012). Phylogenetic analysis was performed using molecular evolutionary genetic analysis (MEGA) version 6.06 (Tamura et al. 2013) with the list of hits from EzBioCloud 16S rRNA database. Scanning electron microscopy (SEM) observation of strain S5.2 was performed with osmium tetroxide fixing and ethanol dehydration procedures followed by viewing using a SEM TM3030 (Hitachi, Japan) device in accordance to Lau et al. (2014) with minor modification.

### Phenotype microarray analysis

The carbon utilization profile of strain S5.2 was assessed using the 96-well PM1 and PM2A plates (Biolog, USA). In brief, the overnight cultured bacterial colonies were inoculated into IF-0a GN/GP base inoculating fluid (Biolog, USA) to reach 85% transmittance (T) according to the manufacturer’s protocol. Aliquots (100 µl) of cell suspension and 1× Biolog redox dye mix A were inoculated into each well of the plates respectively, followed by incubation at 28 °C. The utilization and growth of 192 different carbon substrates from the plates were monitored for 48 h with readings taken at 15 min intervals.

The kinetic information was recorded and quantified using OmniLog OL_FM_12 kinetic software (Biolog, USA) followed by data analysis (Bochner et al. 2001). In the event of bacterial growth, photographic readings of colour intensity resulted in dye reduction were represented in OmniLog units (OU) (Khatri et al. 2013). The threshold for positive bacterial growth was established at 100 OU, calculated with the subtraction of maximum growth value with the first reading (0 h).

### Genome sequencing, assembly and annotation

Genomic DNA of *P*. *mendocina* S5.2 was purified using MasterPure DNA Purification Kit (Epicentre, Illumina Inc., Madison, Wisconsin, USA) followed by purity measurement and quantification using NanoDrop 2000™ spectrophotometer (Thermo Scientific, MA, USA) and Qubit 2.0^®^ fluorometer (Life Technologies, MA, USA), respectively. Sequencing library was prepared from purified genomic DNA according to the guidelines of Template Preparation Kit (Pacific Biosciences, Inc., CA) with library size targeted at 20 kb. Sequence collection was then carried out in 2 SMRT cells using P6/C4 chemistry on a PacBio RS II platform (See-Too et al. 2016).

De novo assembly of the long reads was performed using the Hierarchical Genome Assembly Process (HGAP) version 2.0 using the PacBio SMRT portal. Gepard dotplot program (Krumsiek et al. 2007) was employed to verify the circularity of the resulted contigs followed by circularization of overlapping ends using minimus2 pipeline in AMOS software package (Treangen et al. 2011). Genome annotation was subsequently performed using Rapid Prokaryotic Genome Annotation (PROKKA)(Seemann 2014).

### Bioinformatics analyses

Candidate genes potentially involved in the metabolism of carbon sources were validated using the rapid annotation subsystems technology (RAST) server (Aziz et al. 2008) and NCBI prokaryotic genome annotation pipeline (PGAP). Subsequently, MEGA version 6.06 was also employed for multiple sequence alignment of the amino acids. Additionally, pairwise average nucleotide identity (ANI) analysis using strain S5.2 and other relative *Pseudomonas* spp. was conducted using JSpeciesWS (http://jspecies.ribohost.com/jspeciesws/) with above cutoff more than 95% (Richter et al. 2016). Lastly, the synteny and comparative analysis on the genes of interest were conducted based on SyntTax web server using default parameter followed by manual verification (Oberto 2013).

## Results

### Properties of *P*. *mendocina* strain S5.2

SEM showed that cells of strain S5.2 were 1.5–2.5 µm in length and 0.8–1.0 µm in width (Additional file 1: Figure S1). Pairwise similarity analysis on EzBioCloud database followed by phylogenetic analysis using complete nucleotide sequences of 16S rRNA showed that strain S5.2 was closely related to *P*. *mendocina* NBRC 14162^T^ (Additional file 2: Figure S2). This observation was later verified with ANIb analysis (Additional file 3: Table S1).

### Organic acid utilization profiling

Among the 192 putative carbon sources tested, *P*. *mendocina* S5.2 was able to utilize 58 compounds as sole carbon sources. With reference to the grapevine and root exudates related organic acids, positive utilization was detected for l-lactic acid, succinic acid, d-malic acid, l-malic acid, d, l-malic acid, citric acid and fumaric acid, with average OU values at 243 ± 55 and growth value up to 293 with citric acid as sole carbon (Table 1). The inability of the strain to utilize oxalic, sorbic and all enantiomers of tartaric acids with average growth values of 26 ± 7 was also observed (Table 1).

**Table 1:** Utilization of grapevine and root exudates related compounds as sole carbon source by *P*. *mendocina* strain S5.2

Microplate	Plate position	Carbon source	Growth observed	Omnilog unit (OU)
PM1	A5	Succinic acid	+	288
	B9	l-Lactic acid	+	257
	C3	d, l-Malic acid	+	255
	C7	d-Fructose	−	36
	C9	α-d-Glucose	+	233
	D11	Sucrose	−	32
	E2	m-Tartaric acid	−	17
	F2	Citric acid	+	293
	F5	Fumaric acid	+	256
	G11	d-Malic acid	+	126
	G12	l-Malic acid	+	270
PM2A	C2	l-Glucose	−	22
	D12	Butyric acid	+	159
	F2	Malonic acid	+	206
	F4	Oxalic acid	−	25
	F9	Sorbic acid	−	21
	F11	d-Tartaric acid	−	18
	F12	l-Tartaric acid	−	21

### Genome properties

The genome of *P*. *mendocina* S5.2 contained a ~ 5.12 Mb circular chromosome and a ~ 0.25 Mb megaplasmid that was later designated as pPME5. They were assembled in a single contig each with average coverages of 181.55× and 206.96× obtained for the chromosome and pPME5 plasmid, respectively. The mean G+C content of the chromosome (62.4%) was found to be higher than that of pPME5 (54.7%). Among the 4641 predicted protein coding genes, a total of 3747 (80.7%) could be associated with clear functions. In contrast, only 24 out of 319 (7.5%) open reading frames of pPME5 were predicted to have known functions (Table 2).

**Table 2:** General features of the *P*. *mendocina* strain S5.2 genome predicted in PGAP

Genetic elements	Chromosome	Plasmid pPME5
Size (bp)	5,120,146	252,328
G+C content (%)	62.4	54.7
Protein coding genes	4641	319
Genes with predicted functions	3747	24
rRNA genes (5S, 16S, 23S)	4, 4, 4	0, 0, 0
tRNA genes	66	4
Other RNA genes	4	0

### Genomic features associated with organic acid utilization

**Fig. 1:**
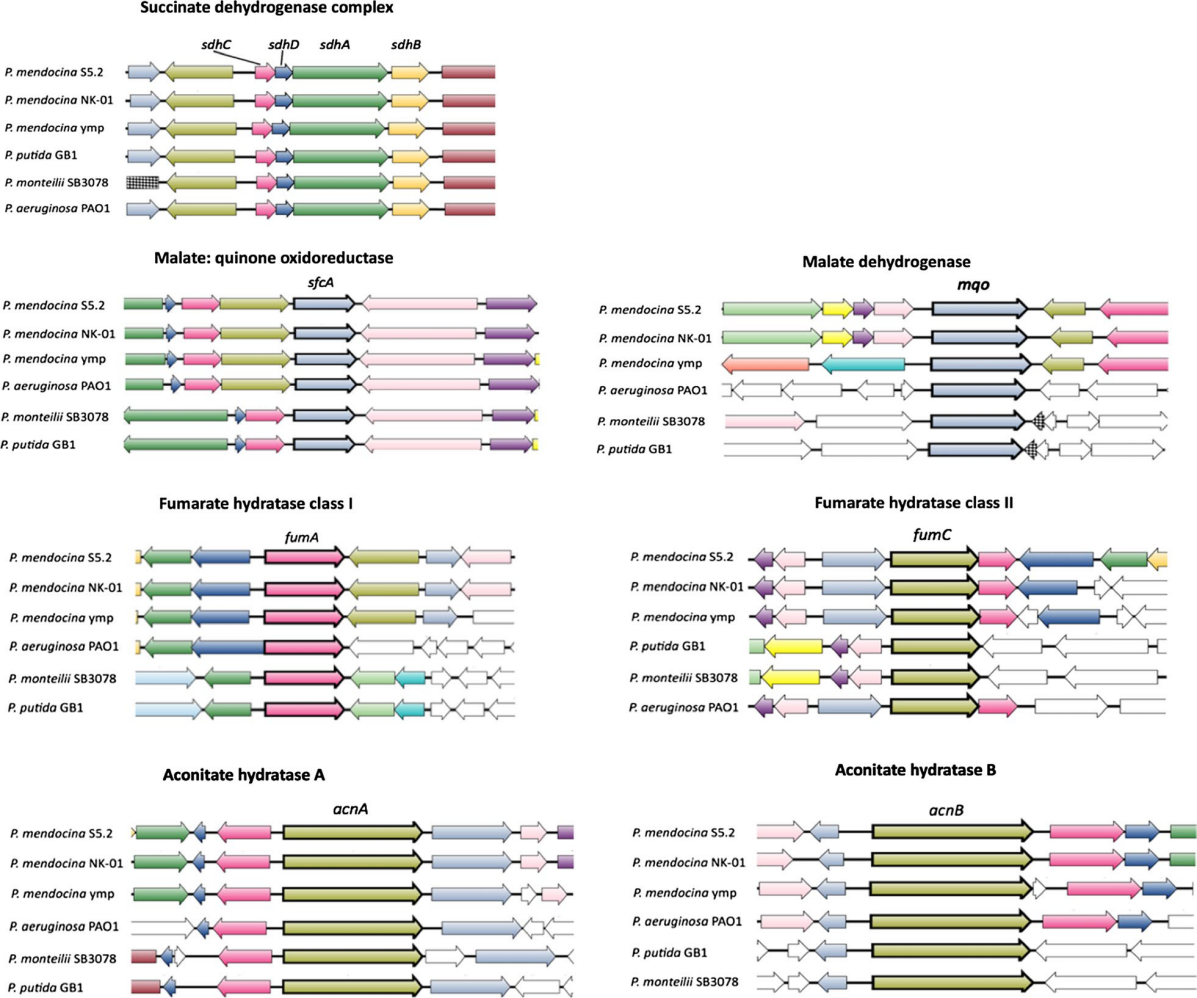
Organization and synteny of putative gene clusters and operons correlated to succinic, malic, fumaric and citric acids utilization of *P*. *mendocina* S5.2

The collective genomic and phenotypic information in the study has enabled the identification of genes for the specific carbon utilization. The circular chromosome of *P*. *mendocina* strain S5.2 was found to harbor a series of orthologous genes and operons associated with utilization traits of grapevine and root related organic acids (Fig. 1; Table 1).

For utilization of succinic acid, the *sdhCDAB* operon encoding succinate dehydrogenase (SDH) complex, a tricarboxylic (TCA) cycle enzyme was identified (Table 3). The reported catalysis of SDH involves the oxidation of succinate to fumarate, coupled with the reduction of ubiquinone to ubiquinol (Ackrell et al. 1992; Westenberg and Guerinot 1999).

**Table 3:** Identified ORFs in the chromosome associated with organic acid and sugar metabolism of *P*. *mendocina* strain S5.2

Locus tag	Annotation/predicted Role	ORF	Position in genome	Size (bp)	Orientation
Succinic acid (Succinate + ubiquinone → fumarate + ubiquinol)					
DW68_012890	Succinate dehydrogenase (iron–sulfur subunit)	*sdhB*	2,790,535–2,791,242	708	←
DW68_012895	Succinate dehydrogenase (flavoprotein subunit)	*sdhA*	2,791,254–2,793,026	1773	←
DW68_012900	Succinate dehydrogenase (cytochrome b small subunit)	*sdhD*	2,793,030–2,793,398	369	←
DW68_012905	Succinate dehydrogenase (cytochrome b560 subunit)	*sdhC*	2,793,392–2,793,766	374	←
Lactic acid					
DW68_005140	d-Lactate dehydrogenase	*dld*	1,090,088–1,091,806	1719	←
DW68_005145	l-Lactate dehydrogenase	*lldD*	1,091,811–1,092,950	1140	←
DW68_005150	l-Lactate permease	*lldP*	1,093,035–1,094,726	1692	←
DW68_005155	Lactate responsive regulator	*lldR*	1,095,022–1,095,789	768	→
Malic acid (malate ⇌ oxaloacetate)					
DW68_020815	Malate dehydrogenase	*sfcA*	4,486,244–4,487,512	1269	←
Malic acid (malate → oxaloacetate)					
DW68_008120	Malate:quinone oxidoreductase	*mqo*	1,719,586–1,721,193	1608	→
Fumaric acid (fumarate ⇌ Malate)					
DW68_007085	Fumarate hydratase class I	*fumA*	1,503,228–1,504,751	1524	←
DW68_014875	Fumarate hydratase class II	*fumC*	3,205,005–3,206,399	1395	→
Citric acid (citrate → isocitrate)					
DW68_010115	Aconitate hydratase A	*acnA*	2,182,629–2,185,370	2742	→
DW68_013280	Aconitate hydratase B	*acnB*	2,870,427–2,873,027	2601	→

**Fig. 2:**
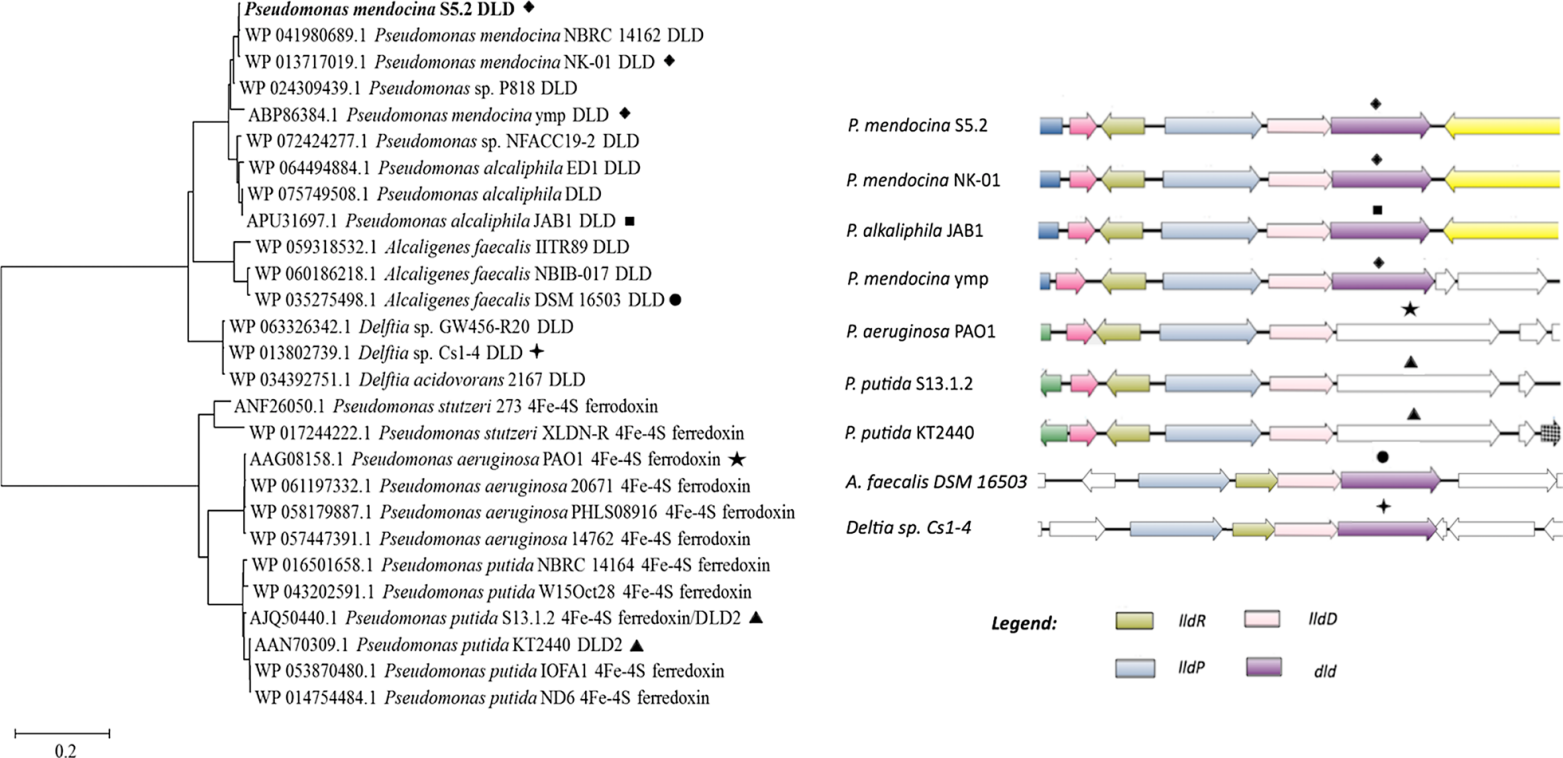
*Left* phylogenetic analysis of putative d-lactate dehydrogenase (DLD) of strain S5.2 relative to other *Pseudomonas*, *Alcaligenes faecalis* and *Deftia* spp. *Right* comparison and synteny of *lldRPD* and *dld* genes among different strains. Positions of the compared DLD related genes in the phylogenetic tree were marked in respective shapes

Besides, an operon associated with lactic acid utilization encoding putative NAD-independent lactate dehydrogenase (iLDH) activity was also present. This enzyme possibly catalyzes the oxidation of lactate to pyruvate, a feature essential for most lactate-utilizing bacteria (Diez-Gonzalez et al. 1995; Gao et al. 2015; Gibello et al. 1999; Goffin et al. 2004). Components of this operon also included a lactate permease (*lldP*), l-lactate dehydrogenase (*lldD*) and d-lactate dehydrogenase gene (*dld)* indicating that strain S5.2 could degrade both lactate enantiomers (Table 3). Adjacent to these genes was *lldR* that encoded a transcriptional regulator possibly controlling the expression of the above-described genes. Comparative analysis with other *Pseudomonas* strains showed a high degree of synteny for the *lldRPD* genes (Fig. 2). Although sharing the identical flavin adenine dinucleotide (FAD)-binding site with all compared strains, sequence similarity search showed that the *dld* of strain 5.2 was similar only to those of some *Pseudomonas* spp., *Alcaligenes* spp., and *Deftia* spp. strains.

The growth of *P*. *mendocina* strain S5.2 was observed in the presence of malic acid enantiomers. Two distinctive genes encoding malate dehydrogenase (SfcA) and malate quinone oxidoreductase (MQO) were found (Table 3). These enzymes shared a general activity catalyzing the oxidation of malate to oxaloacetate. SfcA is a cytoplasmic malate dehydrogenase that reversibly oxidizes malate and is a principal enzyme in the TCA cycle that requires nicotinamide adenine dinucleotide (NAD) as an electron acceptor (van der Rest et al. 2000). Alternatively, MQO is FAD-dependent, membrane associated protein that irreversibly oxidizes malate and donates electrons to quinones of the electron transfer chain (Kather et al. 2000).

Following the fumaric acid utilization phenotype, the genome also harbored genes encoding two classes of fumarate hydratase, namely FumA and FumC (Table 3). FumA is a class I fumarase that catalyzes the interconversion of fumarate to malate and requires iron–sulfur (Fe–S) cluster as a cofactor (Chenier et al. 2008; Flint et al. 1992). In contrast, FumC does not rely on Fe–S clusters for hydration of fumarate to malate (Chenier et al. 2008; Hassett et al. 1997). Coexistence of the isoenzymes in the genome may permit the strain to better survive under iron deficiency conditions. Genes for aconitate hydratases, AcnA and AcnB were also detected (Table 3) in the genome ensuing the proficient metabolism of citric acid reported above. Acn proteins catalyze the stereospecific isomerization of citrate to isocitrate and require Fe–S as an enzyme cofactor, as does FumA (Beinert and Kennedy 1993; Mailloux et al. 2006).

## Discussion

The present work has demonstrated the carbon utilization signature of *P*. *mendocina* strain S5.2 in relation with in silico identification of genomic features associated with grapevine organic acid utilization. Tartaric, malic, oxalic, lactic, citric and succinic acids are among the main organic acids detected in grapevines and root exudates across various genotypes (Andersen and Brodbeck 1989; Cançado et al. 2009; Dharmadhikari 1994; Kliewer 1966; Li et al. 2013; López-Rayo et al. 2015; Mato et al. 2007). The illustrated utilization profile of these organic acids by *P*. *mendocina* strain S5.2 has provided new insights into the diversity of carbon utilization by *P*. *mendocina*. Subsequently, the gene-trait matching approach has demonstrated that these organic acids are catabolic substrates for the strain, involving the concerted actions of enzymes featured in TCA cycle and possibly other metabolic pathways. Such profile may also reflect on the specific nutrient requirements of the strain towards the given niche of the rhizosphere, in which the composition of the deposited exudates was affected by the aforementioned environmental factors (Nihorimbere et al. 2011; Singh and Mukerji 2006).

As an update on the previously reported draft genome of *P*. *mendocina* strain S5.2, the availability of complete genome sequences has allowed accurate definition of gene coordinates and recognition of paralogous gene families. Of note, the unique feature of DLD gene of strain S5.2 was highlighted in this study through amino acid sequence alignment and phylogenetic analysis. Despite sharing a high degree of synteny and similarity for genes encoding LldR, LldP and LldD, the DLD of strain S5.2 did not group with the putative DLD2/ferrodoxin of most *Pseudomonas* spp. Instead, the DLD of strain S5.2 shared higher similarity withs DLD of *P*. *alcaliphila* and other betaproteobacteria strains (*Alcaligenes faecelis* and *Deftia* sp.) (Fig. 2). Essentially, phylogenomic analysis conducted by Gomila and coworkers (2015) showed that *P*. *mendocina* and *P*. *alcaliphila* were in fact clustered under *P*. *oleovorans* group which is closely related to *P*. *aeruginosa* group. Hence such differential grouping of DLD even among the closely related groups of *Pseudomonas* might represent distintive catalytic mechanism required for d-lactate metabolism.

On the other hand, the identification of several isoenzymes has indicated differential catabolic preferences in relation with changes in environmental factors. For instance, the identification of Fe–S independent isoenzymes for utilization of malic acid, MQO and fumaric acid, FumC could be an indication of the essentiality of these enzymes during iron deficiency events. In addition, the absence of MQO has been shown to impede utilization of acetate, ethanol and acyclic terpenes in *Pseudomonas* strains, hence implying the essentiality of MQO in the metabolic versatility of strain S5.2 (Förster-Fromme and Jendrossek 2005; Kretzschmar et al. 2002).

Interestingly, strain S5.2 was shown to exhibit some resistance to copper (Chong et al. 2012). It is also possible to relate the carbon utilization profile with heavy metal resistance traits. Indeed, various carbon sources can serve as an effective electron donor for a given metal resistance, as observed with the reduction rate of hexavalent chromium [Cr(VI)] and trivalent iron [Fe(III)] by *Cellulomonas* sp. ES6 in presence of molasses versus pure sucrose (Field et al. 2013). Also, a combination of various carbon sources was required for chromium reduction by *Klebsiella* sp. PB6 and by a bacterial consortium in contaminated sediments (Smith et al. 2002; Wani and Omozele 2015). Given the heavy metal resistant traits displayed by *Pseudomonas* strains in previous studies (Chan et al. 2016; Chong et al. 2012, 2016), the preferences in utilizing the specific carbon compounds for in situ remediation of copper and other metal ions for instance in vineyard environments should be conducted in future research.

In conclusion, the present work has demonstrated the carbon utilization signature of *P*. *mendocina* strain S5.2, together with in silico identification of genomic features associated with grapevine organic acid utilization. Taken together with the relative cooper resistance identified earlier (Chong et al. 2012), this work demonstrates the remarkable adaptation of *P. mendocina* strain S5.2 to the grapevine and vineyards environment.

## Additional files

**Additional file 1: Figure S1.** Scanning electron micrograph of *P*. *mendocina* strain S5.2. Cells of strain S5.2 measured at the size of 1.5–2.5 µm in length and 0.8–1.0 µm in width.

**Additional file 2: Figure S2.** Phylogenetic tree highlighting the positions of *P*. *mendocina* strain S5.2 relative to other strains within the *Pseudomonas* genus.

**Additional file 3: Table S1.** ANIb result (%) generated from JSpeciesWS (http://jspecies.ribohost.com/jspeciesws/) using strain S5.2 and other relative *Pseudomonas* spp. strains. The green colour indicates above cutoff (>95%).

## Abbreviations

ANI: average nucleotide identity
DLD: d-lactate dehydrogenase
FAD: flavin adenine dinucleotide
Fe–S: iron–sulfur
HGAP: Hierarchical Genome Assembly Process
iLDH: NAD-independent lactate dehydrogenases
LB: Luria–Bertani
LDH: lactate dehydrogenase
lldD: l-lactate dehydrogenase
lldP: lactate permease
MEGA: molecular evolutionary genetic analysis
MQO: malate quinone oxidoreductase
NAD: nicotinamide adenine dinucleotide
OU: OmniLog units
PGAP: prokaryotic genome annotation pipeline
PROKKA: rapid prokaryotic genome annotation
RAST: rapid annotation subsystems technology
SDH: succinate dehydrogenase
SEM: scanning electron microscopy
SfcA: malate dehydrogenase
SMRT: single molecule real-time
TCA: tricarboxylic

## References

[CR1] Ackrell BAC, Johnson MK, Gunsalus RP, Cecchini G, Mueller F (1992). Structure and function of succinate dehydrogenase and fumarate reductase. Chemistry and biochemistry of flavoenzymes.

[CR2] Andersen PC, Brodbeck BV (1989). Diurnal and temporal changes in the chemical profile of xylem exudate from *Vitis rotundifolia*. Physiol Plant.

[CR3] Archana S, Prabakar K, Raguchander T, Hubballi M, Valarmathi P, Prakasam V (2011). Defense responses of grapevine to *Plasmopara viticola* induced by azoxystrobin and *Pseudomonas fluorescens*. Int J Agric Sustain.

[CR4] Aziz RK, Bartels D, Best AA, Dejongh M, Disz T, Edwards RA, Formsma K, Gerdes S, Glass EM, Kubal M, Meyer F, Olsen GJ, Olson R, Osterman AL, Overbeek RA, McNeil LK, Paarmann D, Paczian T, Parrello B, Pusch GD, Reich C, Stevens R, Vassieva O, Vonstein V, Wilke A, Zagnitko O (2008). The RAST server: rapid annotations using subsystems technology. BMC Genom.

[CR5] Bais HP, Weir TL, Perry LG, Gilroy S, Vivanco JM (2006). The role of root exudates in rhizosphere interactions with plants and other organisms. Annu Rev Plant Biol.

[CR6] Beinert H, Kennedy MC (1993). Aconitase, a two-faced protein: enzyme and iron regulatory factor. FASEB J.

[CR7] Bochner BR, Gadzinski P, Panomitros E (2001). Phenotype microarrays for high-throughput phenotypic testing and assay of gene function. Genome Res.

[CR8] Brimecombe MJ, de Leij FA, Lynch JM, Pinton R, Varanini Z, Nannipieri P (2001). The effect of root exudates on rhizosphere microbial populations. The rhizosphere: biochemistry and organic substances at the soil–plant interface.

[CR9] Cançado GMA, Ribeiro AP, Piñeros MA, Miyata LY, Alvarenga ÂA, Villa F, Pasqual M, Purgatto E (2009). Evaluation of aluminium tolerance in grapevine rootstocks. Vitis.

[CR10] Chan K-G, Chong T-M, Adrian T-G-S, Kher HL, Grandclément C, Faure D, Yin W-F, Dessaux Y, Hong K-W (2016). *Pseudomonas lini* strain ZBG1 revealed carboxylic acid utilization and copper resistance features required for adaptation to vineyard soil environment: a draft genome analysis. J Genom.

[CR11] Chenier D, Beriault R, Mailloux R, Baquie M, Abramia G, Lemire J, Appanna V (2008). Involvement of fumarase C and NADH oxidase in metabolic adaptation of *Pseudomonas fluorescens* cells evoked by aluminum and gallium toxicity. Appl Environ Microbiol.

[CR12] Chong TM, Yin W-F, Mondy S, Grandclément C, Dessaux Y, Chan K-G (2012). Heavy-metal resistance of a France vineyard soil bacterium, *Pseudomonas mendocina* strain S5.2, revealed by whole-genome sequencing. J Bacteriol.

[CR13] Chong TM, Yin W-F, Chen J-W, Mondy S, Grandclément C, Faure D, Dessaux Y, Chan K-G (2016). Comprehensive genomic and phenotypic metal resistance profile of *Pseudomonas putida* strain S13.1.2 isolated from a vineyard soil. AMB Express.

[CR14] Dharmadhikari M (1994). Composition of grapes. Vineyard Vintage View Mo State Univ.

[CR15] Diez-Gonzalez F, Russell JB, Hunter JB (1995). The role of an NAD-independent lactate dehydrogenase and acetate in the utilization of lactate by *Clostridium acetobutylicum* strain P262. Arch Microbiol.

[CR16] FeZ Haichar, Marol C, Berge O, Rangel-Castro JI, Prosser JI, Balesdent J, Heulin T, Achouak W (2008). Plant host habitat and root exudates shape soil bacterial community structure. ISME J.

[CR17] Field EK, Gerlach R, Viamajala S, Jennings LK, Peyton BM, Apel WA (2013). Hexavalent chromium reduction by *Cellulomonas* sp. strain ES6: the influence of carbon source, iron minerals, and electron shuttling compounds. Biodegradation.

[CR18] Flint DH, Emptage MH, Guest JR (1992). Fumarase a from *Escherichia coli*: purification and characterization as an iron–sulfur cluster containing enzyme. Biochemistry.

[CR19] Förster-Fromme K, Jendrossek D (2005). Malate: quinone oxidoreductase (MqoB) is required for growth on acetate and linear terpenes in *Pseudomonas citronellolis*. FEMS Microbiol Lett.

[CR20] Gao C, Wang Y, Zhang Y, Lv M, Dou P, Xu P, Ma C (2015). NAD-independent l-lactate dehydrogenase required for l-lactate utilization in *Pseudomonas stutzeri* A1501. J Bacteriol.

[CR21] Gibello A, Collins MD, Domínguez L, Fernández-Garayzábal JF, Richardson PT (1999). Cloning and analysis of the l-lactate utilization genes from *Streptococcus iniae*. Appl Environ Microbiol.

[CR22] Goffin P, Lorquet F, Kleerebezem M, Hols P (2004). Major role of NAD-dependent lactate dehydrogenases in aerobic lactate utilization in *Lactobacillus plantarum* during early stationary phase. J Bacteriol.

[CR23] Gomila M, Peña A, Mulet M, Lalucat J, García-Valdés E (2015). Phylogenomics and systematics in *Pseudomonas*. Front Microbiol.

[CR24] Hassett DJ, Howell ML, Sokol PA, Vasil ML, Dean GE (1997). Fumarase C activity is elevated in response to iron deprivation and in mucoid, alginate-producing *Pseudomonas aeruginosa*: cloning and characterization of fumC and purification of native fumC. J Bacteriol.

[CR25] Karagöz K, Ateş F, Karagöz H, Kotan R, Çakmakçı R (2012). Characterization of plant growth-promoting traits of bacteria isolated from the rhizosphere of grapevine grown in alkaline and acidic soils. Eur J Soil Biol.

[CR26] Kather B, Stingl K, van der Rest ME, Altendorf K, Molenaar D (2000). Another unusual type of citric acid cycle enzyme in *Helicobacter pylori*: the malate: quinone oxidoreductase. J Bacteriol.

[CR27] Khatri B, Fielder M, Jones G, Newell W, Abu-Oun M, Wheeler PR (2013). High throughput phenotypic analysis of *Mycobacterium tuberculosis* and *Mycobacterium bovis* strains’ metabolism using biolog phenotype microarrays. PLoS ONE.

[CR28] Kim O-S, Cho Y-J, Lee K, Yoon S-H, Kim M, Na H, Park S-C, Jeon YS, Lee J-H, Yi H, Won S, Chun J (2012). Introducing EzTaxon-e: a prokaryotic 16S rRNA gene sequence database with phylotypes that represent uncultured species. Int J Syst Evol Microbiol.

[CR29] Kliewer WM (1966). Sugars and organic acids of *Vitis vinifera*. Plant Physiol.

[CR30] Kretzschmar U, Rückert A, Jeoung J-H, Görisch H (2002). Malate: quinone oxidoreductase is essential for growth on ethanol or acetate in *Pseudomonas aeruginosa*. Microbiology.

[CR31] Krumsiek J, Arnold R, Rattei T (2007). Gepard: a rapid and sensitive tool for creating dotplots on genome scale. Bioinformatics.

[CR32] Lau YY, Yin W-F, Chan K-G (2014). *Enterobacter asburiae* strain L1: complete genome and whole genome optical mapping analysis of a quorum sensing bacterium. Sensors.

[CR33] Li K, Guo XW, Xie HG, Guo Y, Li C (2013). Influence of root exudates and residues on soil microecological environment. Pak J Bot.

[CR34] López-Rayo S, Foggia MD, Moreira ER, Donnini S, Bombai G, Filippini G, Pisis A, Rombolà AD (2015). Physiological responses in roots of the grapevine rootstock 140 Ruggeri subjected to Fe deficiency and Fe-heme nutrition. Plant Physiol Biochem.

[CR35] Mailloux RJ, Hamel R, Appanna VD (2006). Aluminum toxicity elicits a dysfunctional TCA cycle and succinate accumulation in hepatocytes. J Biochem Mol Toxicol.

[CR36] Mato I, Suárez-Luque S, Huidobro JF (2007). Simple determination of main organic acids in grape juice and wine by using capillary zone electrophoresis with direct UV detection. Food Chem.

[CR37] Mondy S, Lenglet A, Beury-Cirou A, Libanga C, Ratet P, Faure D, Dessaux Y (2014). An increasing opine carbon bias in artificial exudation systems and genetically modified plant rhizospheres leads to an increasing reshaping of bacterial populations. Mol Ecol.

[CR38] Nihorimbere V, Ongena M, Smargiassi M, Thonart P (2011). Beneficial effect of the rhizosphere microbial community for plant growth and health. Biotechnol Agron Soc Environ.

[CR39] Oberto J (2013). SyntTax: a web server linking synteny to prokaryotic taxonomy. BMC Bioinformatics.

[CR40] Richter M, Rosselló-Móra R, Glöckner FO, Peplies J (2016). JSpeciesWS: a web server for prokaryotic species circumscription based on pairwise genome comparison. Bioinformatics.

[CR41] Seemann T (2014). Prokka: rapid prokaryotic genome annotation. Bioinformatics.

[CR42] See-Too W-S, Convey P, Pearce DA, Lim YL, Ee R, Yin W-F, Chan K-G (2016). Complete genome of *Planococcus rifietoensis* M8^T^, a halotolerant and potentially plant growth promoting bacterium. J Biotechnol.

[CR43] Singh G, Mukerji KG, Mukerji KG, Manoharachary C, Singh J (2006). Root exudates as determinant of rhizospheric microbial biodiversity. Microbial activity in the rhizoshere.

[CR44] Smith WA, Apel WA, Petersen JN, Peyton BM (2002). Effect of carbon and energy source on bacterial chromate reduction. Bioremediat J.

[CR45] Tamura K, Stecher G, Peterson D, Filipski A, Kumar S (2013). MEGA6: molecular evolutionary genetics analysis version 6.0. Mol Biol Evol.

[CR46] Treangen TJ, Sommer DD, Angly FE, Koren S, Pop M (2011). Next generation sequence assembly with AMOS. Curr Protoc Bioinform.

[CR47] Uren NC, Pinton R, Varanini Z, Nannipieri P (2007). Types, amounts, and possible functions of compounds released into the rhizosphere by soil-grown plants. The rhizosphere: biochemistry and organic substances at the soil–plant interface.

[CR48] van der Rest ME, Frank C, Molenaar D (2000). Functions of the membrane-associated and cytoplasmic malate dehydrogenases in the citric acid cycle of *Escherichia coli*. J Bacteriol.

[CR49] Wani PA, Omozele AB (2015). Cr(VI) removal by indigenous *Klebsiella* species PB6 isolated from contaminated soil under the influence of various factors. Curr Res Bacteriol.

[CR50] Westenberg DJ, Guerinot ML (1999). Succinate dehydrogenase (Sdh) from *Bradyrhizobium japonicum* is closely related to mitochondrial Sdh. J Bacteriol.

