# Supplementary figures and images for: Phenotypic and genomic survey on organic acid utilization profile of Pseudomonas mendocina strain S5.2, a vineyard soil isolate
Source: AMB Express. 2017 Jun 26;7:138. doi: 10.1186/s13568-017-0437-7 (PMC5484659; doi:10.1186/s13568-017-0437-7)

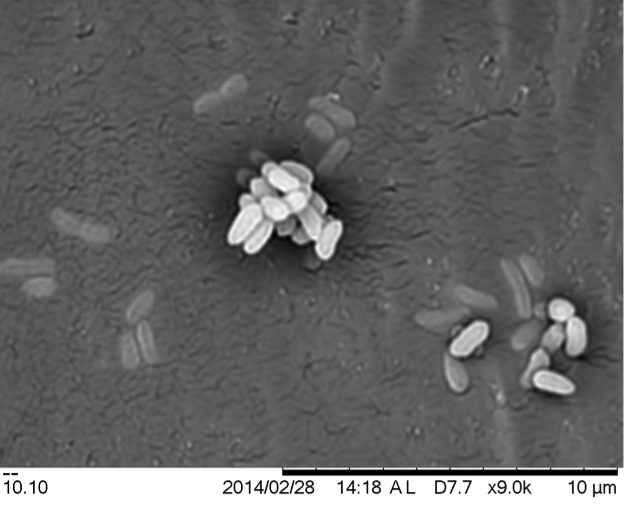

Supplement: Supplementary file 1 — Additional file 1: Figure S1. Scanning electron micrograph of P. mendocina strain S5.2. Cells of strain S5.2 measured at the size of 1.5–2.5 µm in length and 0.8–1.0 µm in width. [file 13568_2017_437_MOESM1_ESM.png]

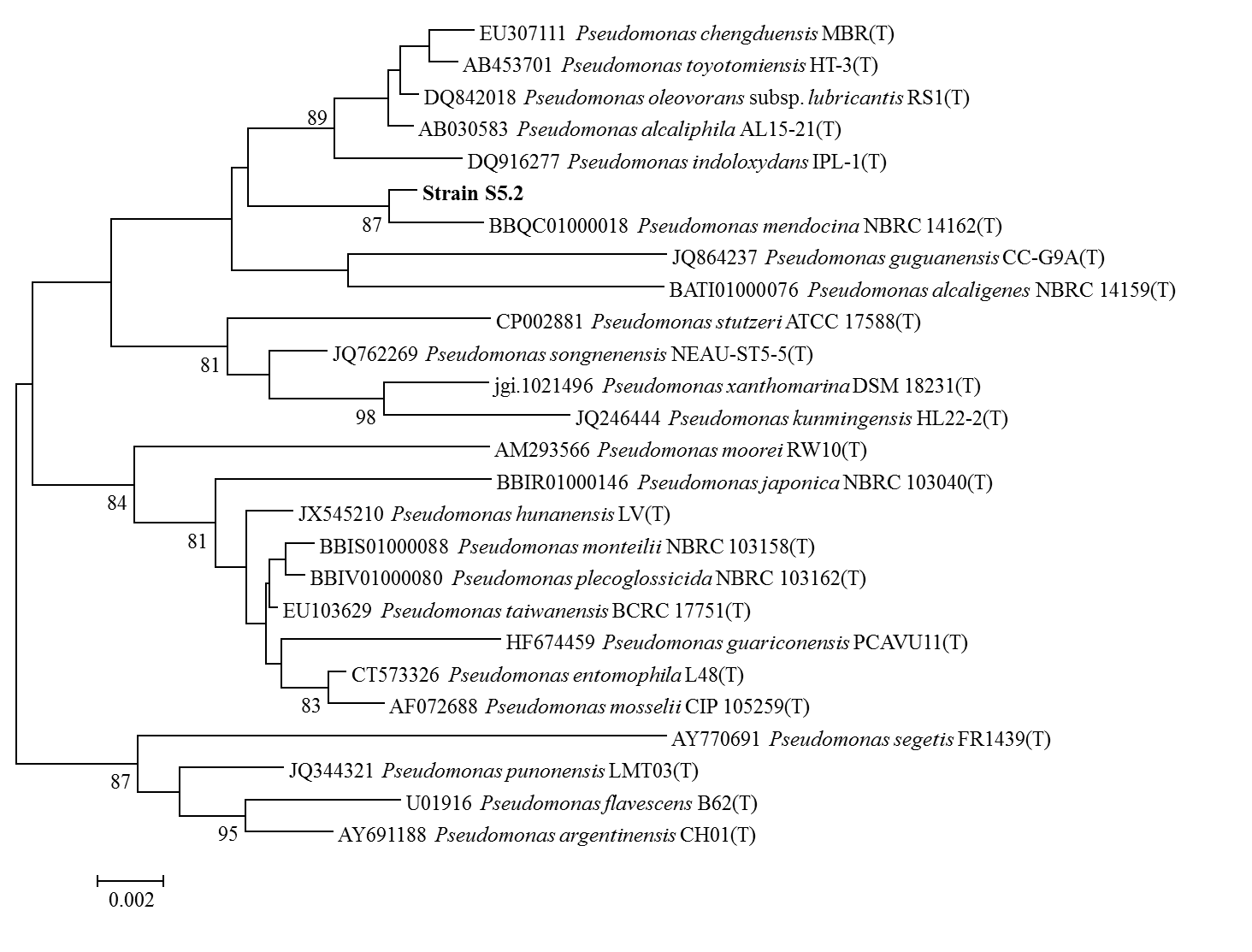

Supplement: Supplementary file 2 — Additional file 2: Figure S2. Phylogenetic tree highlighting the positions of P. mendocina strain S5.2 relative to other strains within the Pseudomonas genus. [file 13568_2017_437_MOESM2_ESM.png]
