# Supplementary material for: Phenotypic and genomic survey on organic acid utilization profile of Pseudomonas mendocina strain S5.2, a vineyard soil isolate
Source: AMB Express. 2017 Jun 26;7:138. doi: 10.1186/s13568-017-0437-7 (PMC5484659; doi:10.1186/s13568-017-0437-7)
Supplement: Supplementary file 3 — Additional file 3: Table S1. ANIb result (%) generated from JSpeciesWS (http://jspecies.ribohost.com/jspeciesws/) using strain S5.2 and other relative Pseudomonas spp. strains. The green colour indicates above cutoff (>95%). [file 13568_2017_437_MOESM3_ESM.docx]

**Table S1**

ANIb result (%) generated from JSpeciesWS (<http://jspecies.ribohost.com/jspeciesws/>) using strain S5.2 and other relative *Pseudomonas* spp. strains. The green colour indicates above cutoff (> 95%)

|  | **1** | **2** | **3** | **4** | **5** | **6** | **7** | **8** | **9** | **10** | **11** | **12** | **13** | **14** | **15** |
| --- | --- | --- | --- | --- | --- | --- | --- | --- | --- | --- | --- | --- | --- | --- | --- |
| 1 |  | 97.66 | 97.58 | 88.04 | 77.73 | 75.94 | 75.71 | 75.49 | 73.98 | 75.89 | 87.95 | 75.83 | 75.51 | 74.99 | 76.13 |
| 2 | 97.72 |  | 97.76 | 88.00 | 77.83 | 75.85 | 75.50 | 75.31 | 73.92 | 75.78 | 88.00 | 75.63 | 75.35 | 74.83 | 75.96 |
| 3 | 97.45 | 97.59 |  | 88.00 | 77.83 | 75.95 | 75.59 | 75.44 | 73.81 | 75.82 | 87.85 | 75.84 | 75.47 | 75.08 | 76.10 |
| 4 | 87.88 | 87.87 | 87.91 |  | 78.05 | 76.17 | 75.83 | 75.66 | 73.96 | 76.08 | 96.72 | 76.09 | 75.83 | 75.46 | 76.49 |
| 5 | 77.22 | 77.20 | 77.26 | 77.44 |  | 76.85 | 76.40 | 76.18 | 74.58 | 75.63 | 77.37 | 76.40 | 76.32 | 75.83 | 75.90 |
| 6 | 75.19 | 75.17 | 75.14 | 75.48 | 76.54 |  | 80.32 | 80.19 | 73.71 | 74.47 | 75.48 | 80.30 | 80.11 | 79.31 | 74.68 |
| 7 | 75.33 | 75.25 | 75.26 | 75.66 | 76.63 | 80.90 |  | 87.13 | 73.81 | 74.69 | 75.62 | 84.42 | 84.31 | 83.66 | 74.96 |
| 8 | 75.37 | 75.30 | 75.24 | 75.52 | 76.32 | 80.57 | 86.95 |  | 73.53 | 74.59 | 75.50 | 84.67 | 84.15 | 83.77 | 74.55 |
| 9 | 73.89 | 73.85 | 73.79 | 73.88 | 74.92 | 74.07 | 73.92 | 73.74 |  | 73.22 | 73.94 | 73.77 | 73.57 | 73.01 | 73.54 |
| 10 | 76.24 | 76.25 | 76.09 | 76.41 | 76.31 | 75.28 | 74.98 | 75.27 | 73.53 |  | 76.85 | 75.09 | 74.90 | 74.45 | 85.88 |
| 11 | 87.70 | 87.75 | 87.66 | 96.55 | 77.88 | 75.86 | 75.54 | 75.56 | 73.60 | 76.17 |  | 75.76 | 75.54 | 75.05 | 76.63 |
| 12 | 75.33 | 75.27 | 75.44 | 75.61 | 76.60 | 80.49 | 84.14 | 84.59 | 73.50 | 74.21 | 75.52 |  | 99.05 | 86.34 | 74.35 |
| 13 | 75.29 | 75.16 | 75.21 | 75.58 | 76.58 | 80.33 | 84.09 | 84.09 | 73.42 | 74.29 | 75.43 | 98.87 |  | 88.77 | 74.58 |
| 14 | 74.71 | 74.62 | 74.81 | 75.16 | 76.11 | 79.65 | 83.70 | 83.84 | 72.82 | 73.98 | 74.88 | 89.40 | 88.85 |  | 74.13 |
| 15 | 76.12 | 76.09 | 76.05 | 76.53 | 76.42 | 75.13 | 74.74 | 74.58 | 73.60 | 85.70 | 76.94 | 74.67 | 74.55 | 74.16 |  |

| **1** | Strain S5.2 (This study) | **9** | *P*. *oleovorans* MOIL14HWK12 |
| --- | --- | --- | --- |
| **2** | *P*. *mendocina* NBRC 14162(T) | **10** | *P*. *xanthomarina* S11 |
| **3** | *P*. *mendocina* NK-01 | **11** | *P*. *toyotomiensis* KF710 |
| **4** | *P*. *alcaliphila* 34 | **12** | *P*. *taiwanensis* SJ9 |
| **5** | *P*. *alcaligenes* OT 69 | **13** | *P*. *plecoglossicida* NyZ12 |
| **6** | *P*. *japonica* NBRC 103040(T) | **14** | *P*. *monteilii* DSM 14164(T) |
| **7** | *P*. *entomophila* L48(T) | **15** | *P*. *stutzeri* KOS6 |
| **8** | *P*. *mosselii* SJ10 |  |  |
